# Quantitative metrics for evaluating surgical dexterity using virtual reality simulations

**DOI:** 10.1371/journal.pone.0318660

**Published:** 2025-03-03

**Authors:** Mingyu Wu, Cham Ying Kit, Eileen Lee Ming Su, Che Fai Yeong, Siti Nor Zawani Ahmmad, William Holderbaum, Chenguang Yang

**Affiliations:** 1 Jiaxing Key Laboratory of Industrial Intelligence and Digital Twin, Jiaxing Vocational and Technical College, Jiaxing, Zhejiang, China; 2 Faculty of Electrical Engineering, Universiti Teknologi Malaysia, Skudai, Johor, Malaysia; 3 Instrumentation and Control Engineering, Universiti Kuala Lumpur, MITEC, Persiaran Sinaran Ilmu, Bandar Seri Alam, Johor, Malaysia; 4 Department of Engineering, Manchester Metropolitan University, Manchester, United Kingdom; 5 Department of Computer Science, University of Liverpool, Liverpool, United Kingdom; National Trauma Research Institute, AUSTRALIA

## Abstract

This study develops and evaluates quantitative metrics to assess surgical dexterity within virtual reality (VR) simulations to enhance surgical training and performance. By employing advanced VR technology, this research systematically investigates the influence of controlled experimental factors—posture, handedness, and visual magnification—on surgical performance. The impact of human factors such as surgical specialty, experience, and lifestyle factors like sleep and caffeine consumption on surgical dexterity is also analyzed. The findings reveal that seated posture, dominant hand usage, and enhanced visual magnification significantly improve surgical precision and eﬃciency. Contrary to common beliefs, lifestyle factors such as sleep duration and coffee consumption showed minimal impact on performance metrics. The study highlights the potential of VR simulations to provide a controlled, replicable, and safe environment for surgical training, emphasizing the importance of personalized training protocols that cater to individual surgeon’s needs. The insights from this research advocate for integrating quantitative, objective metrics in surgical training programs to refine and accelerate dexterity acquisition, ultimately aiming to improve patient outcomes and surgical care.

## Introduction

Surgical competence relies on visuo-spatial awareness and fine motor skills, essential for performing complex operations with minimal risk and optimal outcomes [1]. Traditionally, these skills are acquired through practice and mentorship in clinical settings [2,3]. However, traditional methods often struggle to replicate the complexity of real surgical environments [4–7].

With the advent of digital technology, virtual reality (VR) has become a transformative tool in surgical training. VR provides a controlled environment where surgical scenarios can be replicated with precision [8–11,20]. It allows for manipulation of variables such as posture, handedness, and visual magnification, which can impact surgical performance [12–14,16,21].

However, current assessment methods in VR settings often lack standardized quantitative metrics. Many evaluations rely on subjective judgments or task completion times, which do not capture the full complexity of surgical dexterity [15,19]. This study proposes objective metrics, including endpoint accuracy, motion path length, and economy of movement, to better assess surgical dexterity. These metrics are used to evaluate performance in different postures, offering insights into their effects on surgical outcomes.

In addition to controllable factors like posture and handedness, non-controllable variables such as years of experience, specialty, and lifestyle habits (e.g., sleep and caffeine intake) are examined to understand their impact on surgical performance. As summarized in Table 1, previous investigations into surgical posture have employed different approaches (e.g., electromyography, self-report questionnaires) to evaluate physical strain or skill performance. However, many of these studies did not objectively link posture-related findings to quantifiable surgical dexterity metrics.

**Table 1:** The different approaches/findings and gaps of study regarding postures during surgery.

Study	Approach/Finding	Gap
Singh et al. (2019) [16]	Postures discomfort measured from questionnaires	Lack of objective measurements on evaluating posture discomfort
Ramakrishnan et al. (2017) [17]	Postures discomfort measured from EMG sensors and questionnaires	Lack of objective measurements to reflect on surgical skills performance
Takayasu et al. (2019) [18]	Experts performed lesser deviations on standing postures compared to novices	Lack of comparison between standing and sitting postures


**Fig 1:**
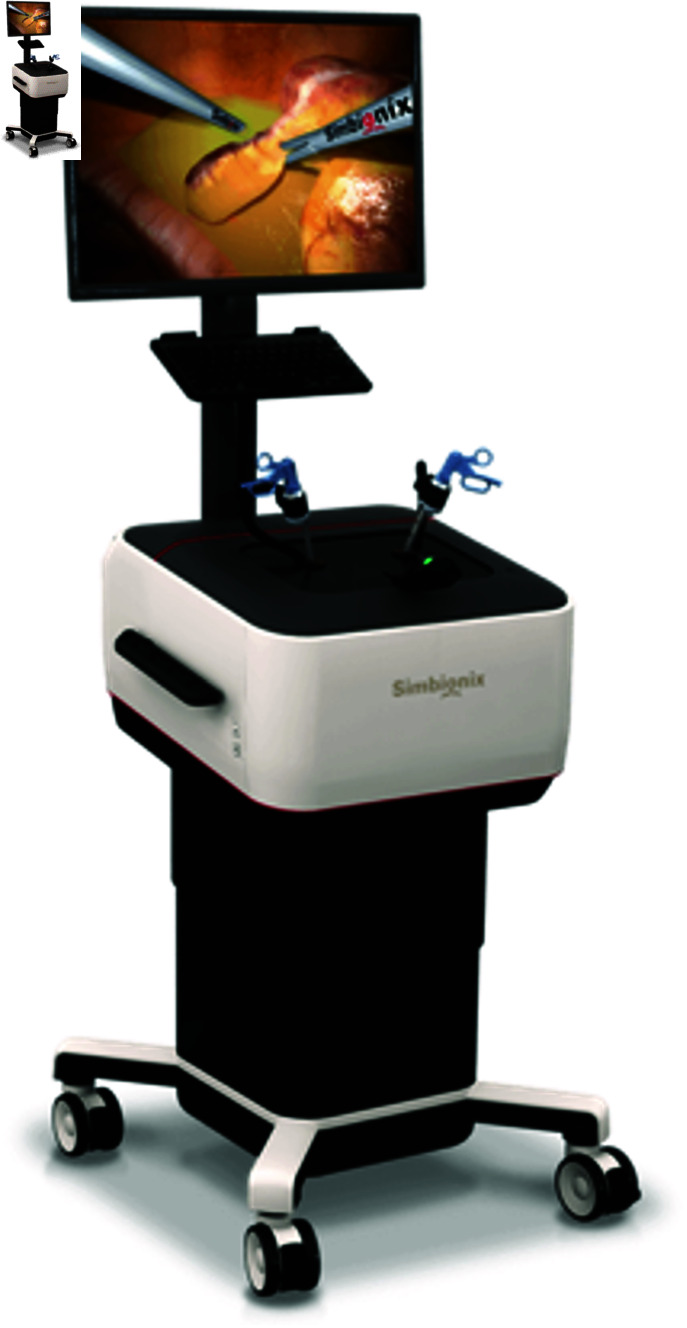
LAP Mentor high-fidelity virtual simulator.

Further analysis of the VR environment setup (Fig 1) includes factors such as handedness and visual magnification, showing performance variations based on these settings.

## Methodology and experimental design

### Research methodology

**Fig 2:**
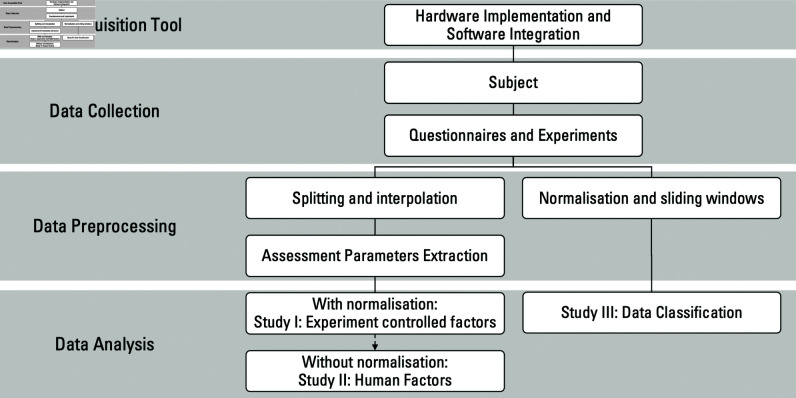
Overall flowchart of research methodology.

This study employed a structured approach combining virtual reality simulation, real-time motion capture, and advanced data analysis techniques. A total of 34 surgeons were recruited from two local hospitals, with informed consent obtained prior to participation. Each participant performed standardized tasks on a high-fidelity VR simulator under controlled conditions, varied by posture (sitting vs. standing), visual magnification (1× vs. 10×), and handedness (dominant vs. non-dominant). The primary data comprised time-series motion tracking of the surgical instruments, from which quantitative metrics such as motion path length, smoothness, and endpoint accuracy were extracted. After data filtering and segmentation, appropriate statistical tests were conducted based on normality assessments to compare performance across the different factors. Fig 2 provides an overview of this methodology, illustrating each stage from participant recruitment to data processing and statistical procedures. The following subsections detail each phase of the study, including participant enrollment, experimental protocol, data processing, and statistical methods.

### Data acquisition tool

#### Hardware implementation.

The hardware implementation for this study consisted of three key components. First, an Acer 3D monitor with an integrated infrared emitter was employed to provide a stereoscopic 3D display, allowing for a more immersive experience during the simulations. The second component was the Sensable PHANTOM Omni devices, chosen for their high precision in capturing 3D positional data, which is critical for accurately recording the movements involved in surgical tasks. Finally, a Dell Alienware M17x served as the computational unit, utilizing its high-performance graphics capabilities to process the acquired data and render the virtual reality environment in real-time.

**Fig 3:**
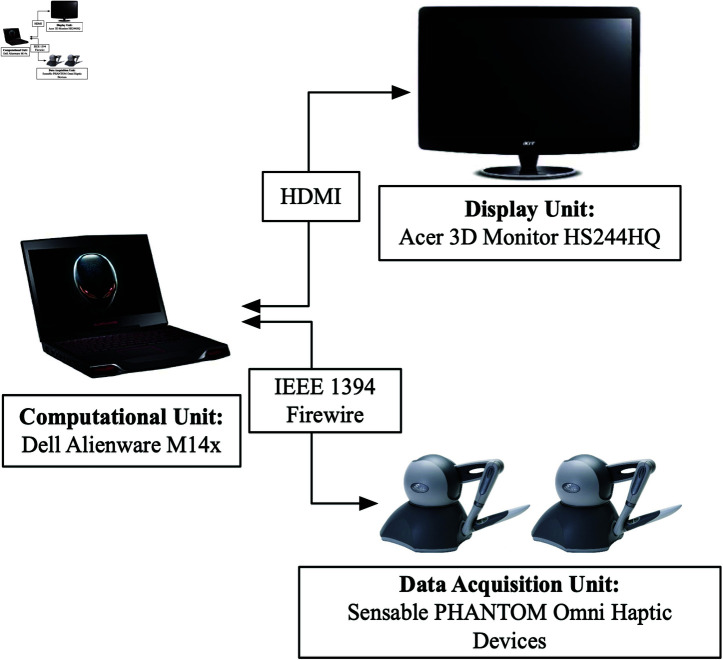
Hardware implementation of the experimental setup.

The complete hardware configuration is illustrated in Fig 3.

#### Software module.

The software module was designed to integrate the hardware components with the virtual environment, ensuring seamless interaction between the user and the simulation. The graphical user interface (GUI), developed using Microsoft Visual Studio C++, enabled intuitive interaction within the VR environment. To handle data synchronization and provide haptic feedback, the software utilized the OpenGL and OpenHaptics libraries, allowing for real-time responsiveness and precision in the simulation.

**Fig 4:**
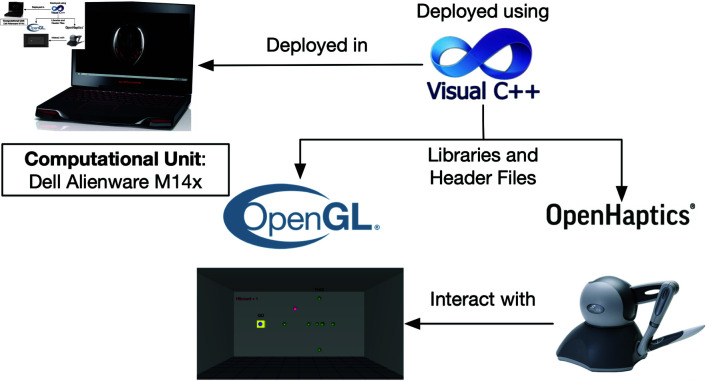
Architecture of software deployment.

The overall software architecture is illustrated in Fig 4.

### Subject recruitment

A total of 34 surgeons from various specialties were recruited from two public hospitals in Johor Bahru, Malaysia, to participate in this study. Each participant was informed about the purpose and procedures of the study, and informed consent was obtained to ensure voluntary participation.

The participants included 19 females and 15 males, ranging in age from 26 to 55 years, with a diverse representation of surgical specialties and years of professional experience. This mix provides a broad base for analyzing the effects of virtual reality training across different demographic and professional backgrounds.

The Table 2 provides a consolidated view of the participants’ demographic and professional data, including gender distribution, age groups, dominant hand, and years of experience:

**Table 2:** Demographic and professional background of participants.

Characteristic	Total	Female	Male	Dominant Hand
Participants	34	19	15	Right: 33, Left: 1
**Age Group (years)**	**26-30**	**31-35**	**36-40**	**41-55**
Number	6	13	7	8
**Years of Experience**	**1-5**	**6-10**	**11+**	
Surgeons	14	14	6	


Table 3 is about lifestyle characteristics such as sleep hours per day, coffee consumption, and video game exposure were also recorded, as these factors can influence cognitive and physical performance:

**Table 3:** Lifestyle characteristics of participants.

Lifestyle Factor	Low	Moderate	High
Sleep Hours per Day	4-6 hours: 14	6-8 hours: 19	>8 hours: 1
Coffee Consumption	None: 12	1-2 cups: 15	<1 cup: 3, 3-4 cups: 4
Video Game Exposure	No: 20	Yes: 12	Maybe: 2


This structured demographic and lifestyle data provides a foundation for assessing the impact of various factors on the effectiveness of VR-based surgical training.

### Data collection protocol

#### Experiment setup.

**Fig 5:**
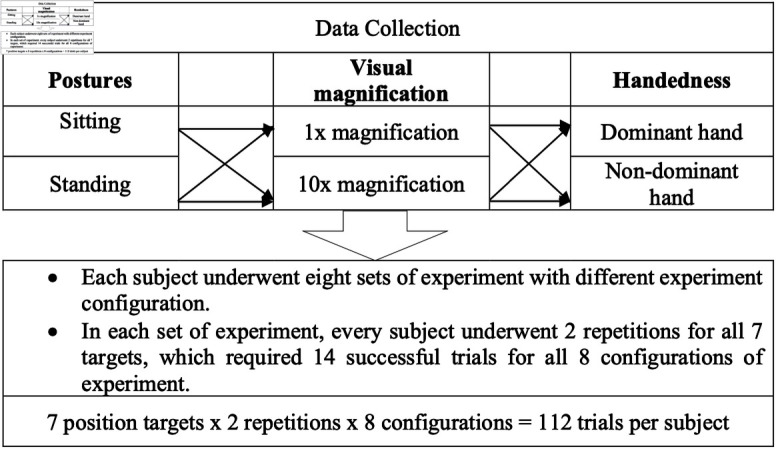
Summary of experimental setups with different controlled configurations.

Subjects performed tasks under varying conditions of posture, visual magnification, and handedness, with each configuration designed to assess different aspects of surgical performance. The setups are summarized in Fig 5.

#### Data collection.

Tooltip positions were captured at millisecond intervals, providing a detailed dataset for movement analysis.

### Data preprocessing and analysis

Eﬃcient data preprocessing is critical for ensuring the integrity and validity of analyses in studies that involve complex and dynamic data, such as those collected from virtual reality surgical simulations. This section describes the methods used to preprocess the data collected from participants performing surgical tasks within a virtual environment.

#### Filtering and normalization.

Data normalization is crucial for ensuring comparability across subjects and sessions. In this study, Z-normalization was applied to each set of movement data to standardize the data distributions, facilitating more accurate comparisons and analyses:


$$\begin{array}{c} Z=\frac{\left(X-\mu \right)}{\sigma } \end{array}$$ (1)

where *X* is the raw data, *μ* is the mean, and *σ* is the standard deviation. This normalization process helps mitigate any discrepancies that arise from individual differences in scale and operational handling, making the data suitable for subsequent statistical evaluations.

#### Segmentation.

The continuous stream of motion data was segmented using a sliding window approach, crucial for analyzing sequences of surgical movements:


$$\begin{array}{c} \text{Segment Length}=2000\text{ points per window} \end{array}$$ (2)

This method divides the motion data into manageable segments, each containing 2000 data points, ensuring that each segment captures a complete action from start to finish. This is particularly useful for isolating specific movements within the surgery simulation, such as reaching for, grasping, and manipulating surgical instruments, allowing for detailed analysis of each phase of the surgical task.

**Fig 6:**
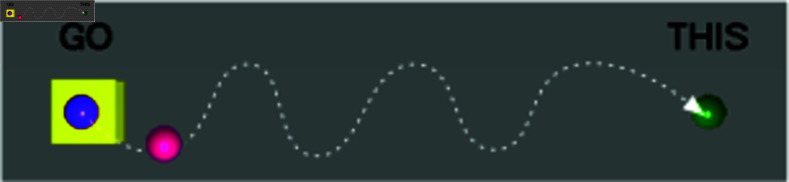
Dynamic motion depicting the cursor moving from the starting point to the target point. This phase assesses the surgeon’s ability to initiate and control movement toward a surgical target, which is critical for tasks that require reaching and positioning within a confined space.

**Fig 7:**
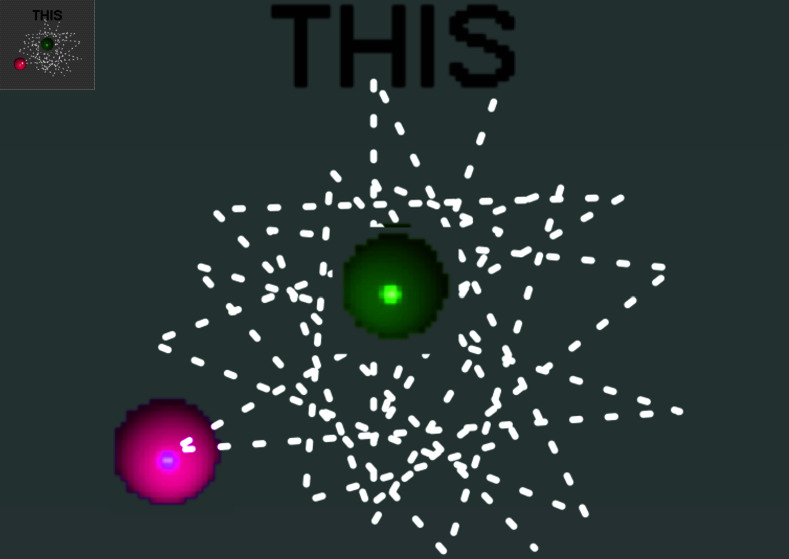
Static motion showing the cursor reaching the target point and remaining steady for 3 seconds. This phase evaluates the surgeon’s capacity for maintaining focus and stability, which is essential for performing precise surgical maneuvers.

Motions captured during the virtual reality simulations were analyzed by categorizing them into dynamic and static phases to assess surgical dexterity finely. Dynamic motions involving the cursor’s movement towards a specific target illustrate the control and eﬃciency of the surgeon’s hand movements and are depicted in Fig 6. Conversely, static motions occur when the cursor is held steady at the target location, demonstrating the surgeon’s ability to maintain precision and stability, as shown in Fig 7. Analyzing these phases separately allows for a nuanced evaluation of different aspects of surgical skill, highlighting the surgeon’s proficiency in executing precise movements and maintaining steadiness under static conditions.

#### Feature extraction.

After segmenting the data, key features such as motion path length, velocity, endpoint accuracy, and motion smoothness were extracted. These features are essential for quantitatively measuring surgical dexterity. For example, motion path length and motion smoothness provide insights into the surgeon’s ability to move eﬃciently and fluidly, while endpoint accuracy and the steadiness of static holds reflect precision and control.

#### Statistical analysis preparation.

Following preprocessing, the data were prepared for statistical analysis. This preparation involved ensuring that all data segments were appropriately aligned and formatted for comparative analysis across different trials and subjects. Statistical methods were then applied to these data to identify significant patterns and differences in performance, which are directly related to the surgeon’s dexterity and competency in virtual reality simulations.

This methodical approach to data preprocessing supports robust and insightful analysis of surgical skills, providing a solid foundation for understanding the nuances of surgical dexterity within a virtual environment.

### Parameter extraction

The motion data collected during the experiments allowed for the extraction of key performance parameters, which were categorized into dynamic and static motion parameters. Dynamic motion parameters included metrics such as motion path length, which was calculated using the following equation:


$$\begin{array}{c} \text{Path Length}=\overset{N-1}{\underset{i=1}{\sum }}\sqrt{{\left(x_{i+1}-x_{i}\right)}^{2}+{\left(y_{i+1}-y_{i}\right)}^{2}+{\left(z_{i+1}-z_{i}\right)}^{2}} \end{array}$$ (3)

This metric provides valuable insight into the eﬃciency of the movements performed during the task. In addition, static motion parameters focused on end-point precision, which is essential for assessing the accuracy of the final positions reached during the procedure. Together, these parameters offer a comprehensive evaluation of the performance, capturing both the eﬃciency and precision of the movements.

### Statistical analysis

In this section, the extracted parameters from the VR simulations were used to conduct a comprehensive statistical analysis to identify which experimental configurations minimized errors and optimized performance, considering the diverse surgical backgrounds of participants. This analysis included normalization and data grouping based on controlled experimental factors and further analysis across various human factors.

#### Data normalization.

To ensure comparability across different experimental settings and participants, each parameter extracted from the data was normalized using the Min-Max normalization technique:


$$\begin{array}{c} X_{\text{norm}}=\frac{X-X_{\text{min}}}{X_{\text{max}}-X_{\text{min}}} \end{array}$$ (4)

where *X*_min_ and *X*_max_ are the minimum and maximum values of the parameter across all settings for each subject. This normalization was crucial for analyzing the influence of experimental control factors such as posture, visual magnification, and handedness.

#### Data grouping.

**Fig 8:**
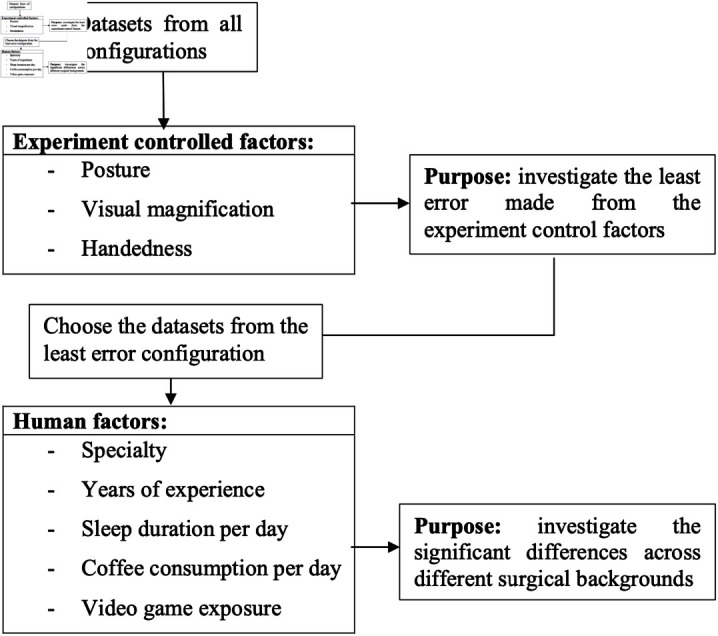
Data grouping based on controlled experimental factors.

The collected data were categorized based on three primary experimental factors: posture (sitting vs. standing), visual magnification (1x vs. 10x), and handedness (dominant vs. non-dominant hand). This grouping allowed for the systematic analysis of how each of these variables impacted surgical dexterity. By focusing on the configuration with the least errors, the study aimed to isolate and assess the influence of each controlled factor. Fig 8 provides an overview of the data grouping process.

#### Statistical methods.

To determine significant differences across the various experimental groups, several statistical tests were employed. The Shapiro-Wilk test was initially used to assess the normality of the data distributions, calculated as follows:


$$\begin{array}{c} W=\frac{{\left(\overset{n}{\underset{i=1}{\sum }}a_{i}x_{\left(i\right)}\right)}^{2}}{\overset{n}{\underset{i=1}{\sum }}{\left(x_{i}-\bar{x}\right)}^{2}} \end{array}$$ (5)

In this equation, *x*_(*i*)_ represents the ordered sample values, while *a*_*i*_ are constants derived from the covariance matrix of the order statistics of a sample of size *n* from a normal distribution.

Based on the results of the normality test, appropriate statistical methods were chosen. For normally distributed data, an Independent Samples *t*-Test was applied when comparing two groups, while an ANOVA was used for comparisons involving more than two groups. In cases where the data did not follow a normal distribution, non-parametric alternatives such as the Mann-Whitney *U* test or the Kruskal-Wallis test were used.

Post-hoc analyses were conducted using Tukey’s Honestly Significant Difference (HSD) test to identify pairwise differences among the groups. The test statistic *q* was calculated as:


$$\begin{array}{c} q=\frac{{\bar{X}}_{i}-{\bar{X}}_{j}}{\sqrt{\text{MSE}∕n}} \end{array}$$ (6)

where ${\bar{X}}_{i}$ and ${\bar{X}}_{j}$ denote the sample means of the groups being compared, MSE represents the mean squared error, and *n* is the sample size for each group.

The aim of these statistical analyses was to identify the experimental setup that consistently produced the lowest error rates. This setup was then further examined in relation to human factors such as surgical specialty, years of experience, and lifestyle habits to assess its overall eﬃcacy.

#### Human factors analysis.

Following the identification of the optimal experimental configuration, the parameters were further analyzed to evaluate their relationship with various human factors. These factors included the surgeon’s specialty (such as general surgery or obstetrics), years of experience (ranging from novice to experienced), and lifestyle habits (such as sleep duration and coffee consumption). The insights gained from this analysis were crucial in developing tailored training programs that accommodate individual differences in background and personal habits, thereby enhancing the educational effectiveness of VR surgical simulations.

## Results

The results section elaborates on the influence of controlled experimental factors and human factors on surgical performance. These factors were assessed using parameters such as motion path length, motion path accuracy, motion path precision, motion smoothness, economy of movement, endpoint accuracy, and endpoint precision. Data were analyzed based on differences in posture, visual magnification, handedness, and various human factors such as specialty, years of experience, sleep, coffee consumption, and video game exposure.

### Effects of experimental controlled factors

#### Posture.

**Fig 9:**
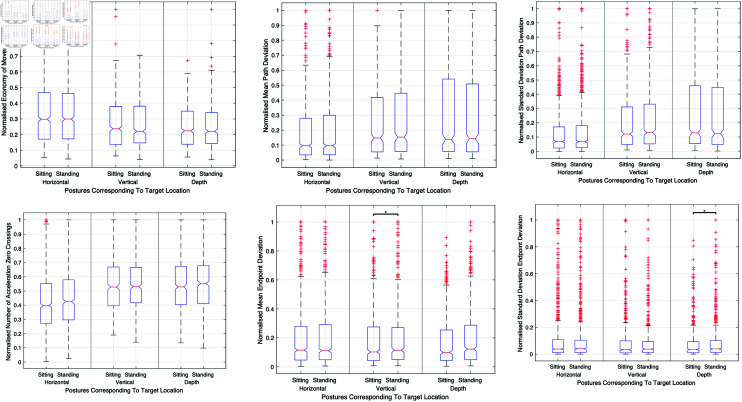
Composite figure illustrating the effects of visual magnification on surgical performance. This figure compares 1x and 10x visual magnifications, showcasing their impact on motion path length, economy of movement, motion path accuracy, precision, and smoothness across various surgical tasks and target locations.

In this study, no significant differences were observed in motion path length, economy of movement, or motion smoothness between sitting and standing postures across various target locations. However, endpoint accuracy and precision varied significantly, with the sitting posture resulting in better endpoint accuracy in vertical target positions. Fig 9 shows a comprehensive comparison of all posture-related performance metrics across different target locations, encapsulating motion path length, economy of movement, accuracy, precision, smoothness, endpoint accuracy, and precision metrics.

#### Visual magnification.

**Fig 10:**
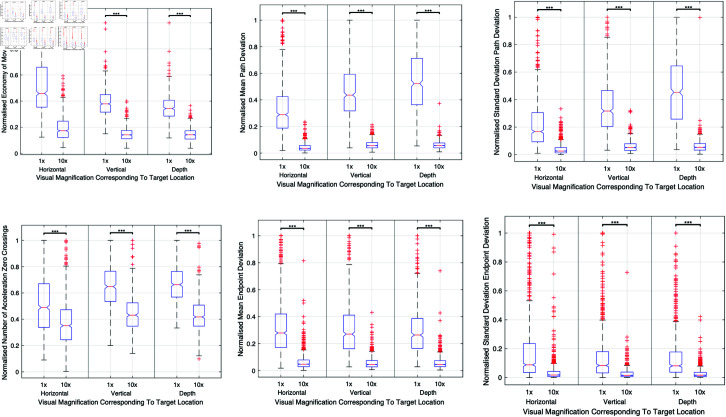
Composite figure illustrating the effects of visual magnification on surgical performance. This figure compares 1x and 10x visual magnifications, demonstrating their impact on motion path length, economy of movement, and motion path precision across different target locations.

The adoption of 10x visual magnification markedly enhanced performance metrics such as motion path length, economy of movement, and motion path precision across all target locations. Fig 10 illustrates the significant improvements achieved with higher magnification, highlighting comparisons across a variety of surgical tasks.

#### Handedness.

**Fig 11:**
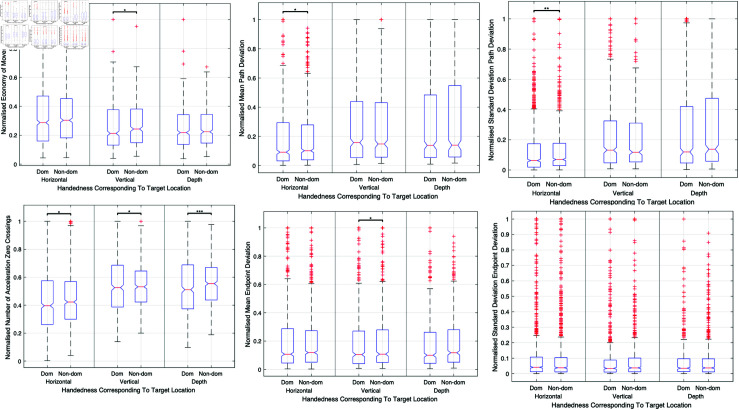
Comparison of surgical performance between dominant and non-dominant hands.

Performance disparities were evident between dominant and non-dominant hands, with the dominant hand typically yielding better results in motion path accuracy, precision, and smoothness, especially in tasks targeting horizontal and vertical planes. Fig 11 combines these findings into a single visualization, providing a clear overview of the comparative performance of dominant versus non-dominant hands.

### Impact of human factors

#### Specialty.

**Fig 12:**
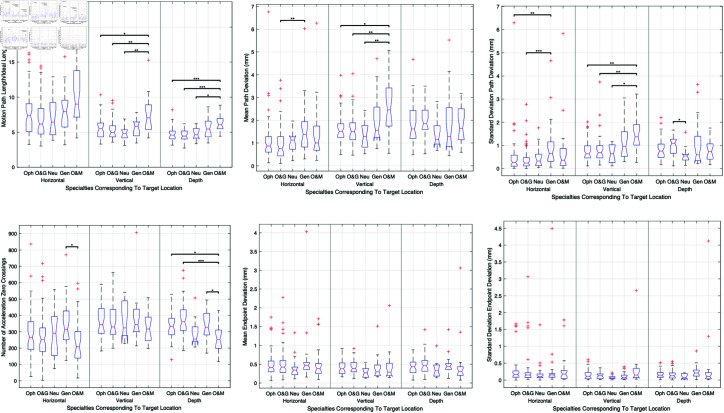
Comprehensive comparison of various performance metrics among different surgical specialties. The metrics include motion path length, economy of movement, motion path accuracy, precision, and smoothness, highlighting significant differences observed particularly in oral and maxillofacial surgery.

Significant differences were noted among specialties, with surgeons from the oral and maxillofacial department exhibiting longer motion paths and lower economy of movement compared to other specialties, especially in vertical and depth target locations. This comprehensive analysis is illustrated in Fig 12, which combines metrics such as motion path length, economy of movement, motion path accuracy, precision, and smoothness across various specialties.

#### Years of experience.

**Fig 13:**
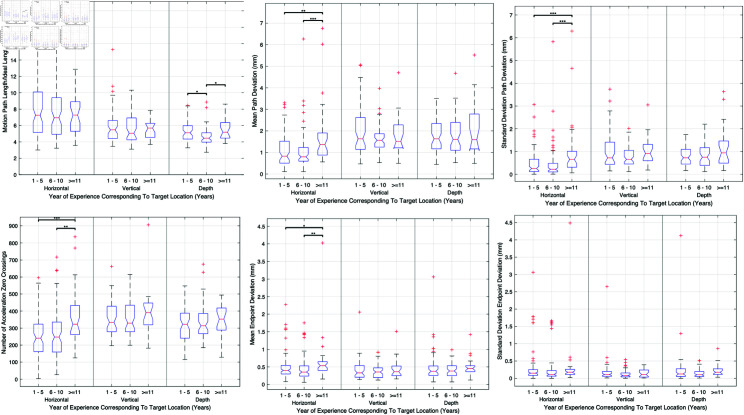
Analysis of surgical performance across different years of experience, emphasizing depth-targeted tasks. This figure integrates data on economy of movement and motion path length, showing enhanced performance in surgeons with 6 to 10 years of experience.

Experience was found to play a crucial role, particularly among surgeons with 6 to 10 years of experience, who demonstrated superior performance(p<0.05), especially in depth-targeted tasks. Fig 13 compiles data on economy of movement and motion path length across experience levels, showing clear trends that emphasize the advantages of moderate experience levels in surgical precision and eﬃciency.

### Lifestyle factors

#### Sleeping hours per day.

**Fig 14:**
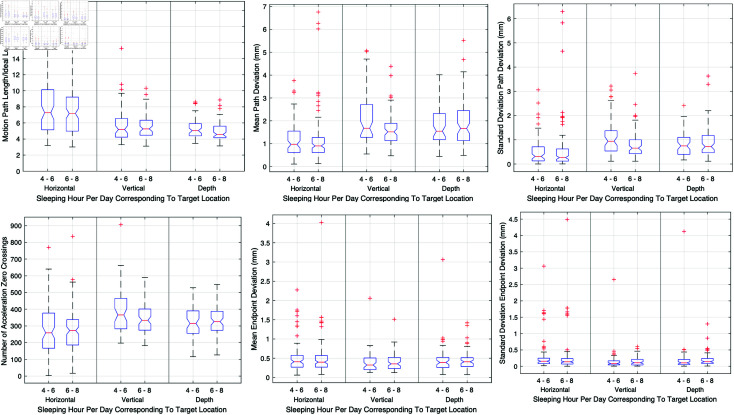
Effects of sleep duration on various performance metrics across different target locations, comparing groups with 4 to 6 hours and 6 to 8 hours of sleep. Metrics include motion path length, economy of movement, accuracy, precision, smoothness, and endpoint metrics.

Investigations into the effects of sleep duration on surgical performance were conducted by comparing two groups: those who slept for 4 to 6 hours and those who slept for 6 to 8 hours nightly. Analysis revealed no significant differences in performance metrics between these groups, suggesting that minor variations in sleep duration within this range do not critically affect surgical capabilities. Fig 14 illustrates these results, showing that the performance in terms of motion path length, economy of movement, accuracy, precision, smoothness, and endpoint metrics remained consistent across both sleep groups.

#### Coffee consumption per day.

**Fig 15:**
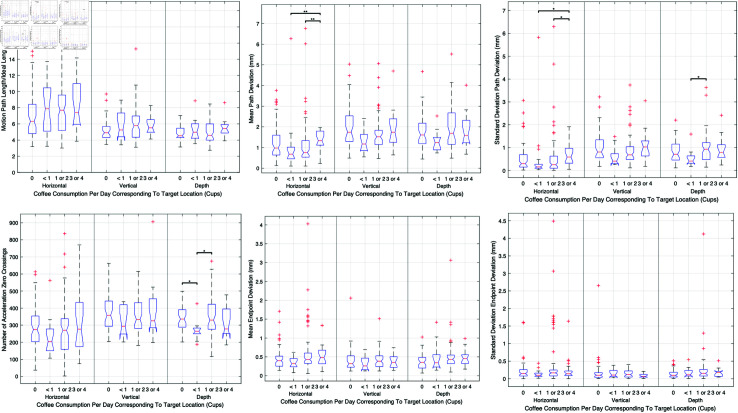
Effects of coffee consumption on surgical performance metrics, aggregating data across various coffee consumption levels and target locations. Differences in performance metrics such as motion path length, economy of movement, and accuracy are highlighted among groups consuming different amounts of coffee daily.

This analysis investigates the influence of coffee consumption on surgical performance. The data reveals no significant impact on motion path length among surgeons with varying daily coffee intakes, as observed during procedures in all target locations. The detailed performance metrics by coffee consumption groups are illustrated in Fig 15, showing comparisons across the groups for metrics such as motion path length, economy of movement, and accuracy.

Despite the general lack of statistically significant differences, subtle variations in performance were noted. For instance, surgeons who consumed no coffee displayed slightly better economy of movement, although these differences were not statistically significant when compared with other groups. Similarly, surgeons consuming three to four cups of coffee daily exhibited some deviations in motion path accuracy, particularly in horizontal target locations, suggesting that higher caffeine intake might affect precision negatively.

In terms of precision, there was a notable difference in the performance of surgeons who consumed three to four cups of coffee daily—they exhibited less precision in motion path compared to those consuming less coffee, particularly in horizontal target locations. This suggests that while coffee consumption does not broadly impact surgical performance, excessive intake could potentially impair specific aspects of surgical precision.

#### Video game exposure.

**Fig 16:**
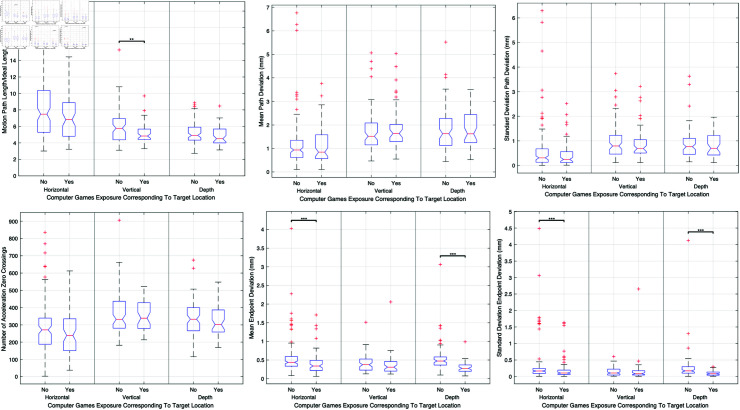
Comparative analysis of the impact of video game exposure on surgical performance, showing metrics such as motion path length, economy of movement, accuracy, precision, smoothness, and endpoint metrics across different target locations for surgeons with and without video game experience.

The relationship between video game exposure and surgical performance was explored, with findings suggesting that surgeons with experience in video games exhibited enhanced performance metrics, especially in tasks requiring precision and accuracy in vertical target locations. Fig 16 displays these advantages, where significant improvements are evident in motion path length, economy of movement, and other performance metrics for surgeons with video game experience, compared to those without such exposure.

## Discussion

This study investigated the impact of controlled experimental factors and human factors, including lifestyle influences, on surgical performance using virtual reality simulations. The findings offer critical insights into optimizing surgical training protocols by highlighting key areas that influence surgical dexterity.

### Controlled experimental factors

**Posture.** Our results demonstrated that a seated posture significantly enhanced endpoint accuracy and precision during surgical tasks. This improvement is likely due to reduced fatigue and discomfort, suggesting that ergonomic considerations in surgical settings can lead to better performance outcomes. These findings align with previous studies emphasizing the benefits of sitting during surgical procedures. For instance, Wauben et al. (2006) [22] reported that surgeons experienced less physical discomfort and improved task performance when seated during laparoscopic surgery. Similarly, Hemal et al. (2001) [23] found that sitting improved the surgeon’s dexterity and reduced fatigue in laparoscopic procedures.

Our study reinforces the importance of ergonomic optimization in surgical environments, suggesting that adopting a seated posture can enhance precision and reduce physical strain, ultimately improving surgical outcomes.

**Handedness.** Surgeons often perform laparoscopic tasks more eﬃciently with their dominant hand, highlighting the necessity of improving non-dominant hand skills through targeted training. For example, Middleton et al. (2013) [24] found that participants who engaged in Nintendo Wii gaming demonstrated improved non-dominant hand performance on a laparoscopic virtual reality simulator. The study revealed significant enhancements in non-dominant hand-eye coordination (p = 0.04) and the economic movement of the left hand during bimanual grasping tasks (p = 0.05). These findings underscore the importance of developing ambidextrous skills, which are critical in minimally invasive surgery where precise bimanual coordination is essential.

Adaptive training strategies that focus on developing proficiency in both hands could mitigate the performance gap between dominant and non-dominant hands. Studies by Molinas et al. (2017) [25] have shown that pre-training in hand-eye coordination with both dominant and non-dominant hands significantly shortens the learning curve for complex laparoscopic tasks, such as intra-corporeal knot tying. Our findings support the incorporation of ambidextrous training modules in surgical education programs to enhance bilateral proficiency.

**Visual magnification.** The use of 10x visual magnification in our study demonstrated improvements in visibility and detail recognition, critical for the precision required in complex surgical procedures. These findings support the integration of advanced visual technologies in surgical practice. Similar results were reported by Safwat et al. (2009) [26], who found that magnification up to ×10 is critical for accurate micromanipulations, enhancing fine motor skills and reducing errors in microsurgical tasks.

Moreover, visual magnification devices can decrease physical strain on surgeons by allowing a more natural posture, thereby combining ergonomic benefits with enhanced visual acuity. The integration of high-definition imaging and magnification technologies should be considered essential components in modern surgical training and practice, as they contribute to improved surgical outcomes and reduced practitioner fatigue.

### Human factors

**Specialty.** Our analysis revealed distinct differences in performance based on surgical specialty. Surgeons from various specialties demonstrated varying levels of performance, particularly in motion path length and economy of movement. These variations suggest that training programs tailored to the unique demands of each surgical specialty could be highly beneficial in enhancing overall surgical proficiency.

This is supported by the work of Grantcharov et al. (2004) [27], who found that specialty-specific training improved skill acquisition and retention in laparoscopic procedures. Tailoring training to address the specific challenges and skill requirements of each specialty may enhance learning eﬃciency and surgical outcomes. Our study emphasizes the importance of customized training curricula that reflect the procedural nuances and dexterity requirements of different surgical fields.

**Experience.** In terms of experience, surgeons with 6 to 10 years of practice exhibited superior performance in specific tasks, highlighting the role of moderate experience in optimizing surgical dexterity. This finding aligns with the concept that accumulated experience and continued practice are critical for skill mastery.

Continuous professional development is essential for maintaining high levels of proficiency. Our findings advocate for the implementation of lifelong learning initiatives and regular skills assessments to ensure that surgeons remain adept with evolving surgical techniques and technologies.

### Lifestyle factors

**Sleep duration.** The analysis of lifestyle factors revealed that within the range of 4 to 8 hours of sleep, surgeons maintained their performance levels. This observation aligns with the notion that medical professionals often adapt to varying sleep schedules due to the demands of their profession. However, it underscores the importance of adequate rest for maintaining optimal performance.

**Caffeine consumption.** Caffeine consumption showed nuanced effects on surgical dexterity. Surgeons who consumed minimal amounts of caffeine exhibited improved motion precision and smoothness. Moderate caffeine consumption is known to improve alertness and cognitive function [28], which may translate into better surgical performance. Our findings support a balanced approach to caffeine consumption among surgeons, advocating for moderation to maximize benefits while minimizing adverse effects.

**Video game exposure.** Additionally, surgeons with video game experience demonstrated superior performance in metrics associated with dexterity and coordination, likely reflecting the dynamics and interactive skills cultivated through gaming. This is consistent with previous research by Schlickum et al. (2009) [29], who found that video game experience correlated with better laparoscopic surgical skills.

The skills developed through video gaming, such as hand-eye coordination, spatial awareness, and rapid decision-making, are transferable to surgical tasks. Incorporating video game-based training modules or serious gaming approaches into surgical education may enhance skill acquisition and proficiency, particularly in younger surgeons who are more accustomed to digital interfaces. Our study highlights the potential of leveraging interactive technologies to enrich surgical training programs.

### Implications for surgical training

These findings underscore the complexity of factors that influence surgical performance and highlight the need for a comprehensive approach to training that considers ergonomic settings, lifestyle factors, innovative educational tools, and individual surgeon characteristics. Virtual reality simulations offer a unique platform to integrate these elements, providing a controlled environment for surgeons to practice and refine their skills.

Future research should expand these observations with a more diverse cohort to generalize these findings and optimize training strategies further. The development of personalized training programs that adapt to the specific needs and backgrounds of individual surgeons could enhance the effectiveness of surgical education, ultimately improving patient outcomes.

### Evaluation of practicality, feasibility, and usefulness

This study demonstrates the practicality and feasibility of using virtual reality (VR) simulations to assess and enhance surgical dexterity. The quantitative metrics we developed provide an objective means of measurement that can be directly integrated into surgical training programs. By employing VR technology, surgeons can practice and refine their skills in a controlled, replicable, and safe environment without risking patient safety.

The usefulness of this approach is evident in its adaptability across different surgical specialties. By adjusting variables such as posture, handedness, and visual magnification, training can be tailored to address the specific needs of various surgical fields. For instance, our findings indicate that training with enhanced visual magnification significantly improves performance metrics in specialties requiring microsurgical skills.

When comparing our results with similar studies within the same or different specialties, the positive impact of VR stimulation on surgical performance is consistently observed. For example, Sutherland et al. [4] highlighted the effectiveness of VR simulations in neurosurgical training, demonstrating improved surgical precision and a shortened learning curve. Our findings align with these results, suggesting that VR-based training can universally enhance surgical dexterity across specialties.

Moreover, as VR technology becomes more accessible and cost-effective, the feasibility of implementing it into surgical training is strengthened. Institutions can integrate these tools into existing curricula, providing trainees with ample opportunities to develop and assess their skills. This approach addresses the limitations of traditional training methods by offering standardized, objective assessments and immediate feedback.

## Conclusions

This study provides a robust framework for evaluating and enhancing surgical dexterity through virtual reality (VR) simulations. The key findings and their implications are summarized as follows:

- **Postural impact:** Adopting a seated posture during surgeries minimizes fatigue and enhances surgical accuracy and precision. This emphasizes the importance of ergonomic considerations in surgical practice to improve performance outcomes.
- **Dominance in handedness:** The performance differences observed between dominant and non-dominant hands highlight the necessity for training programs and technological aids that aim to balance surgical dexterity. Developing ambidextrous skills is crucial for complex procedures requiring high levels of coordination.
- **Visual magnification:** Utilizing high-definition visual magnification significantly improves surgical outcomes by enhancing accuracy, precision, and economy of movement. This underscores the value of integrating advanced visual technologies into surgical training and practice.
- **Experience level:** Surgeons with 6 to 10 years of experience demonstrated superior performance in specific tasks, underscoring the importance of accumulated experience and continuous practice in optimizing surgical dexterity. Targeted training during this critical period could further enhance skill development.
- **Influence of lifestyle factors:** Contrary to expectations, lifestyle factors such as sleep patterns and coffee consumption had minimal impact on surgical performance within the ranges studied. This suggests that while overall health is important, these specific factors may not significantly affect surgical dexterity in controlled settings.
- **Video game exposure:** A positive correlation was observed between video game exposure and enhanced surgical performance, particularly in terms of motion path length and economy of movement. This supports the potential inclusion of video game-like training simulations in surgical education to develop hand-eye coordination and spatial awareness.

The implications of this study are significant for surgical training programs. Incorporating VR simulations that closely mimic actual surgical settings can provide surgeons with a safe and controlled environment to practice and refine their skills. These simulations can be designed to allow variable control over factors such as posture, visual magnification, and handedness, facilitating personalized training that addresses individual needs and specialty requirements.

Given the adaptability of VR technology and the increasing availability of advanced simulation platforms, integrating VR into surgical education is both practical and highly feasible. By comparing our results with similar studies in different specialties, such as the work by Sutherland et al. [4] demonstrating improved precision and shortened learning curves in neurosurgical training, we reinforce the universal applicability of VR simulations in enhancing surgical performance across various fields. This emphasizes the potential for VR technology to become a core component of surgical training programs, ultimately improving patient care and surgical outcomes.

Future research should expand on these findings by including larger and more diverse participant groups to validate and generalize the observed effects globally. Longitudinal studies exploring skill retention and the transfer of simulation-trained skills to clinical practice could provide deeper insights into the progression of surgical expertise. Additionally, expanding the scope to encompass a wider range of surgical tasks and specialties will further validate the effectiveness of this training modality.

In conclusion, this study validates the practicality, feasibility, and usefulness of VR simulations as valuable tools in surgical training. The established quantitative metrics not only provide an objective assessment of surgical dexterity but also promote personalized training approaches that can adapt to individual surgeons’ needs and specialty requirements. By enhancing surgical performance through targeted training and technological integration, we aim to ultimately improve patient outcomes and advance the field of surgical education and practice.

## Supporting information

S1 File game.zip.001 Split-compressed raw data related to games (part 1 of 3).
(ZIP)

S2 File game.zip.002 Split-compressed raw data related to games (part 2 of 3).
(ZIP)

S3 File game.zip.003 Split-compressed raw data related to games (part 3 of 3).
(ZIP)

S4 File coffee.zip Raw data related to coffee.
(ZIP)

S5 File yoe.zip Raw data related to years of experience.
(ZIP)

S6 File role.zip Raw data related to roles.
(ZIP)

S7 File spe.zip.001 Split-compressed data related to specialization (part 1 of 5).
(ZIP)

S8 File spe.zip.002 Split-compressed data related to specialization (part 2 of 5).
(ZIP)

S9 File spe.zip.003 Split-compressed data related to specialization (part 3 of 5).
(ZIP)

S10 File spe.zip.004 Split-compressed data related to specialization (part 4 of 5).
(ZIP)

S11 File spe.zip.005 Split-compressed data related to specialization (part 5 of 5).
(ZIP)

S12 File sleep.zip.001 Split-compressed data related to sleep (part 1 of 5).
(ZIP)

S13 File sleep.zip.002 Split-compressed data related to sleep (part 2 of 5).
(ZIP)

S14 File sleep.zip.003 Split-compressed data related to sleep (part 3 of 5).
(ZIP)

S15 File sleep.zip.004 Split-compressed data related to sleep (part 4 of 5).
(ZIP)

S16 File sleep.zip.005 Split-compressed data related to sleep (part 5 of 5).
(ZIP)

## References

[1] Calne SR. The illustrated history of surgery. London: Routledge; 2018.

[2] Tchantchaleishvili V, Myers PO. Hand laterality and acquired ambidexterity in surgical training. Ann Surg. 2016;264(6):e18–9. doi: 10.1097/SLA.0000000000001951 27537533

[3] Sugden C, Aggarwal R. Assessment and feedback in the skills laboratory and operating room. Surg Clin North Am. 2010;90(3):519–33. doi: 10.1016/j.suc.2010.02.009 20497824

[4] Sutherland LM, Middleton PF, Anthony A, Hamdorf J, Cregan P, Scott D, Maddern GJ. Surgical simulation: a systematic review. Ann Surg. 2006;243(3):291–300. doi: 10.1097/01.sla.0000200839.42045.53 PMC1448942 16495690

[5] Khalifa YM, Bogorad D, Gibson V, Peifer J, Nussbaum J. Virtual reality in ophthalmology training. Surv Ophthalmol. 2006;51(3):259–73. doi: 10.1016/j.survophthal.2006.02.005 16644366

[6] Jaffer A, Bednarz B, Challacombe B, Sriprasad S. The assessment of surgical competency in the UK. Int J Surg. 2009;7(1):12–5. doi: 10.1016/j.ijsu.2008.10.006 19028147

[7] Yiasemidou M, Roberts D, Glassman D, Tomlinson J, Biyani S, Miskovic D. A multispecialty evaluation of thiel cadavers for surgical training. World J Surg. 2017;41(5):1201–7. doi: 10.1007/s00268-016-3868-4 28144746 PMC5394144

[8] Cheng LP, Ofek E, Holz C, Benko H, Wilson AD. Sparse haptic proxy: touch feedback in virtual environments using a general passive prop. In: Proceedings of the 2017 CHI Conference on Human Factors in Computing Systems. New York: Association for Computing Machinery; 2017. p. 3718–28. doi: 10.1145/3025453.3025753

[9] Oropesa I, Sánchez-González P, Lamata P, Chmarra MK, Pagador JB, Sánchez-Margallo JA, et al. Methods and tools for objective assessment of psychomotor skills in laparoscopic surgery. J Surg Res 2011;171(1):e81–95. doi: 10.1016/j.jss.2011.06.034 21924741

[10] Estrada S, O’Malley MK, Duran C, Schulz D, Bismuth J. On the development of objective metrics for surgical skills evaluation based on tool motion. In: 2014 IEEE International Conference on Systems, Man, and Cybernetics (SMC). 2014. 3144–9. doi: 10.1109/smc.2014.6974411

[11] Waterman BR, Martin KD, Cameron KL, Owens BD, Belmont PJ Jr. Simulation training improves surgical proficiency and safety during diagnostic shoulder arthroscopy performed by residents. Orthopedics 2016;39(3):e479–85. doi: 10.3928/01477447-20160427-02 27135460

[12] Su ELM, Win TL, Ang WT, Lim TC, Teo CL, Burdet E. Micromanipulation accuracy in pointing and tracing investigated with a contact-free measurement system. Annu Int Conf IEEE Eng Med Biol Soc. 2009;2009:3960–3. doi: 10.1109/IEMBS.2009.5333665 19964328

[13] Kong X, Yang M, Li X, Ni M, Zhang G, Chen J, et al. Impact of surgeon handedness in manual and robot-assisted total hip arthroplasty. J Orthop Surg Res 2020;15(1):159. doi: 10.1186/s13018-020-01671-0 32316973 PMC7171772

[14] Topalli D, Eyüboğlu BG, Cagiltay NE. Understanding the effect of handedness on both-handed task performance: an experimental study based on a haptic-controlled, simulation-based surgical skill training scenario. Int J Hum–Comput Interact. 2018;35(6):478–82. doi: 10.1080/10447318.2018.1464283

[15] Jardine D, Hoagland B, Perez A, Gessler E. Evaluation of surgical dexterity during the interview day: another factor for consideration. J Grad Med Educ. 2015;7(2):234–7. doi: 10.4300/JGME-D-14-00546.1 26221441 PMC4512796

[16] Singh R, Yurteri-Kaplan LA, Morrow MM, Weaver AL, McGree ME, Zhu X, et al. Sitting versus standing makes a difference in musculoskeletal discomfort and postural load for surgeons performing vaginal surgery. Int Urogynecol J. 2019;30(2):231–7. doi: 10.1007/s00192-018-3619-1 29671032

[17] Ramakrishnan VR, Milam BM. Ergonomic analysis of the surgical position in functional endoscopic sinus surgery. Int Forum Allergy Rhinol. 2017;7(6):570–5. doi: 10.1002/alr.21911 28296272

[18] Takayasu K, Yoshida K, Mishima T, Watanabe M, Matsuda T, Kinoshita H. Upper body position analysis of different experience level surgeons during laparoscopic suturing maneuvers using optical motion capture. Am J Surg. 2019;217(1):12–6. doi: 10.1016/j.amjsurg.2018.06.026 30017308

[19] Ogunyemi D, Alexander C, Tangchitnob E, Kim DS. Mini surgical simulation, role play, and group and behavioral interviews in resident selection. J Grad Med Educ. 2016;8(3):410–6. doi: 10.4300/JGME-D-15-00203.1 27413446 PMC4936861

[20] Benko H, Holz C, Sinclair M, Ofek E. NormalTouch and TextureTouch. In: Proceedings of the 29th Annual Symposium on User Interface Software and Technology. 2016. p. 717–28. doi: 10.1145/2984511.2984526

[21] Schlussel AT, Maykel JA. Ergonomics and musculoskeletal health of the surgeon. Clin Colon Rectal Surg. 2019;32(6):424–34. doi: 10.1055/s-0039-1693026 31686994 PMC6824896

[22] Wauben LSGL, Goossens RHM, van Eijk DJ, Lange JF. Effects of different working positions on performing laparoscopic tasks in a box trainer. Surg Endosc. 2006;20(7):1135–40. doi: 10.1007/s00464-005-0552-9

[23] Hemal AK, Goel RK, Goel A, Gupta NP, Kumar R, Wadhwa SN. Comparative assessment of standing versus sitting position in percutaneous nephrolithotomy for renal calculi. J Endourol. 2001;15(4):359–64. doi: 10.1089/089277901300189300

[24] Middleton KK, Hamilton T, Tsai PC, Middleton DB, Falcone JL, Hamad G. Improved nondominant hand performance on a laparoscopic virtual reality simulator after playing the Nintendo Wii. Surg Endosc. 2013;27(11):4224–31. doi: 10.1007/s00464-013-3077-3 23760943

[25] Molinas CR, Campo R. Dominant hand, non-dominant hand, or both? The effect of pre-training in hand-eye coordination upon the learning curve of laparoscopic intra-corporeal knot tying. Gynecol Surg. 2017;14(1):1–8. doi: 10.1007/s10397-016-1002-2 PMC5570794 28890675

[26] Safwat T, Eldar G, Ross WL. The role of magnification in micromanipulation: improving accuracy and reducing errors. Ann Biomed Eng. 2009;37(12):2676–84. doi: 10.1007/s10439-009-9805-3

[27] Grantcharov TP, Kristiansen VB, Bendix J, Bardram L, Rosenberg J, Funch-Jensen P. Randomized clinical trial of virtual reality simulation for laparoscopic skills training. Br J Surg. 2004;91(2):146-–150. doi: 10.1002/bjs.4403 14760660

[28] Smith A. Effects of caffeine on human behavior. Food Chem Toxicol. 2002;40(9):1243–55. doi: 10.1016/s0278-6915(02)00096-0 12204388

[29] Schlickum MK, Hedman L, Enochsson L, Kjellin A, Felländer-Tsai L. Systematic video game training in surgical novices improves performance in virtual reality endoscopic surgical simulators: a prospective randomized study. World J Surg. 2009;33(11):2360–7. doi: 10.1007/s00268-009-0151-y 19649553

